# Glomerular galactose-deficient IgA1 expression analysis in pediatric patients with glomerular diseases

**DOI:** 10.1038/s41598-020-71101-y

**Published:** 2020-08-20

**Authors:** Shinya Ishiko, Tomoko Horinouchi, Rika Fujimaru, Yuko Shima, Hiroshi Kaito, Ryojiro Tanaka, Shingo Ishimori, Atsushi Kondo, Sadayuki Nagai, Yuya Aoto, Nana Sakakibara, China Nagano, Tomohiko Yamamura, Momoka Yoshimura, Koichi Nakanishi, Junya Fujimura, Naohiro Kamiyoshi, Hiroaki Nagase, Norishige Yoshikawa, Kazumoto Iijima, Kandai Nozu

**Affiliations:** 1 Department of Pediatrics, Kobe University Graduate School of Medicine, 7-5-1 Kusunoki-cho, Chuo-ku, Kobe, Hyogo 650-0017 Japan; 2 Department of Pediatrics, Osaka City General Hospital, Osaka, Japan; 3 Department of Pediatrics, Wakayama Medical University, Wakayama, Japan; 4 Department of Nephrology, Hyogo Prefectural Kobe Children’s Hospital, Kobe, Japan; 5 Department of Pediatrics, Takatsuki General Hospital, Takatsuki, Japan; 6 Department of Child Health and Welfare (Pediatrics), Graduate School of Medicine, University of Ryukyus, Nishihara, Japan; 7 Department of Pediatrics, Kakogawa Central City Hospital, Kakogawa, Japan; 8 Department of Pediatrics, Japanese Red Cross Society Himeji Hospital, Himeji, Japan; 9 Clinical Research Center, Takatsuki General Hospital, Takatsuki, Japan

**Keywords:** IgA nephropathy, Glomerulus, Paediatric kidney disease

## Abstract

Galactose-deficient IgA1 (Gd-IgA1) is important in the pathogenesis of IgA nephropathy (IgAN). A Gd-IgA1-specific monoclonal antibody (KM55) has revealed glomerular Gd-IgA1 deposition solely in patients with IgAN and IgA vasculitis with nephritis (IgAV-N). However, this specificity is controversial and has not been demonstrated in pediatric patients. Here, we conducted double-immunofluorescence staining of IgA and Gd-IgA1 in 60 pediatric patients with various glomerular diseases. We divided patients into four groups: (1) patients with IgAN and IgAV-N (n = 23); (2) patients with immunocomplex-mediated glomerulonephritis accompanied by IgA deposition, including lupus nephritis, membranoproliferative glomerulonephritis, and membranous nephropathy (n = 14); (3) patients with other glomerular diseases involving IgA deposition, including idiopathic nephrotic syndrome (INS), oligomeganephronia, Alport syndrome, dense deposit disease, and crescentic glomerulonephritis (n = 11); and (4) patients with IgA-negative diseases including INS, membranoproliferative glomerulonephritis, membranous nephropathy, oligomeganephronia, Alport syndrome, C3 glomerulonephritis, poststreptococcal acute glomerulonephritis, and hemolytic uremic syndrome (n = 12). KM55 staining revealed Gd-IgA1-positive findings in 23/23 patients in Group 1 and 13/14 patients in Group 2, but not in patients in Groups 3 or 4. Therefore, KM55 may detect incidental IgA deposition in pediatric patients. Gd-IgA1 may be involved in the pathogenesis of these immune-related diseases; alternatively, KM55 may recognize IgA-related immunocomplexes in a non-specific manner.

## Introduction

IgA nephropathy (IgAN) is a common type of primary glomerulonephritis in children. It was initially considered a benign condition, but extended follow-up studies indicated that IgAN was associated with a poor renal prognosis, with a renal survival probability of 79.8% at 20 years for Japanese patients with end-stage kidney disease^1^. In most patients, IgAN is discovered as microscopic hematuria with or without proteinuria and is diagnosed by evaluation of renal biopsy specimens. IgAN is defined by mesangial proliferative nephritis with IgA-dominant or codominant mesangial glomerular deposits.

Considerable advances in understanding the pathogenesis of IgAN have been made over the past two decades, and galactose-deficient IgA1 (Gd-IgA1) has been identified as an essential molecule in this process. However, the detailed underlying molecular mechanisms remain unclear^2–4^. A multi-hit mechanism for the pathogenesis of IgAN is now widely accepted; this mechanism involves increased levels of poorly *O*-galactosylated IgA glycoforms, production of *O*-glycan-specific antibodies, formation of IgA1-containing immune complexes, and resultant mesangial deposition of IgA1-containing immune complexes, which lead to glomerulonephritis^4^. Serum levels of Gd-IgA1 were found to be elevated in 75% of adult and pediatric patients with IgAN based on a *Helix aspersa* agglutinin lectin enzyme-linked immunosorbent assay (ELISA), suggesting that this measurement may be a potential biomarker for the disease^3,5^. A novel lectin-independent ELISA using a specific monoclonal antibody (KM55) that recognizes a hinge region in human Gd-IgA1 (KM55) was recently established^6^; this assay revealed that circulating levels of Gd-IgA1 were elevated in patients with IgAN, consistent with the results of *Helix aspersa* agglutinin lectin ELISA. KM55 can also detect glomerular Gd-IgA1 in tissues using immunohistochemistry techniques; it has identified glomerular Gd-IgA1 deposition as a disease-specific marker of IgAN and IgA vasculitis with nephritis (IgAV-N) in adult patients^7^. Although some studies in adults have investigated glomerular Gd-IgA1 staining with KM55, the specificity of KM55 staining remains controversial^8^. Cassol et al. indicated that Gd-IgA1 staining was present in patients with primary IgAN, as well as in patients with secondary IgAN and staphylococcal infection-associated glomerulonephritis^8^. To the best of our knowledge, no studies of pediatric patients have been published thus far. We aimed to evaluate the specificity of glomerular Gd-IgA1 deposition in children using KM55, thus evaluating its ability to diagnose IgAN and IgAV-N.

## Results

**Figure 1:**
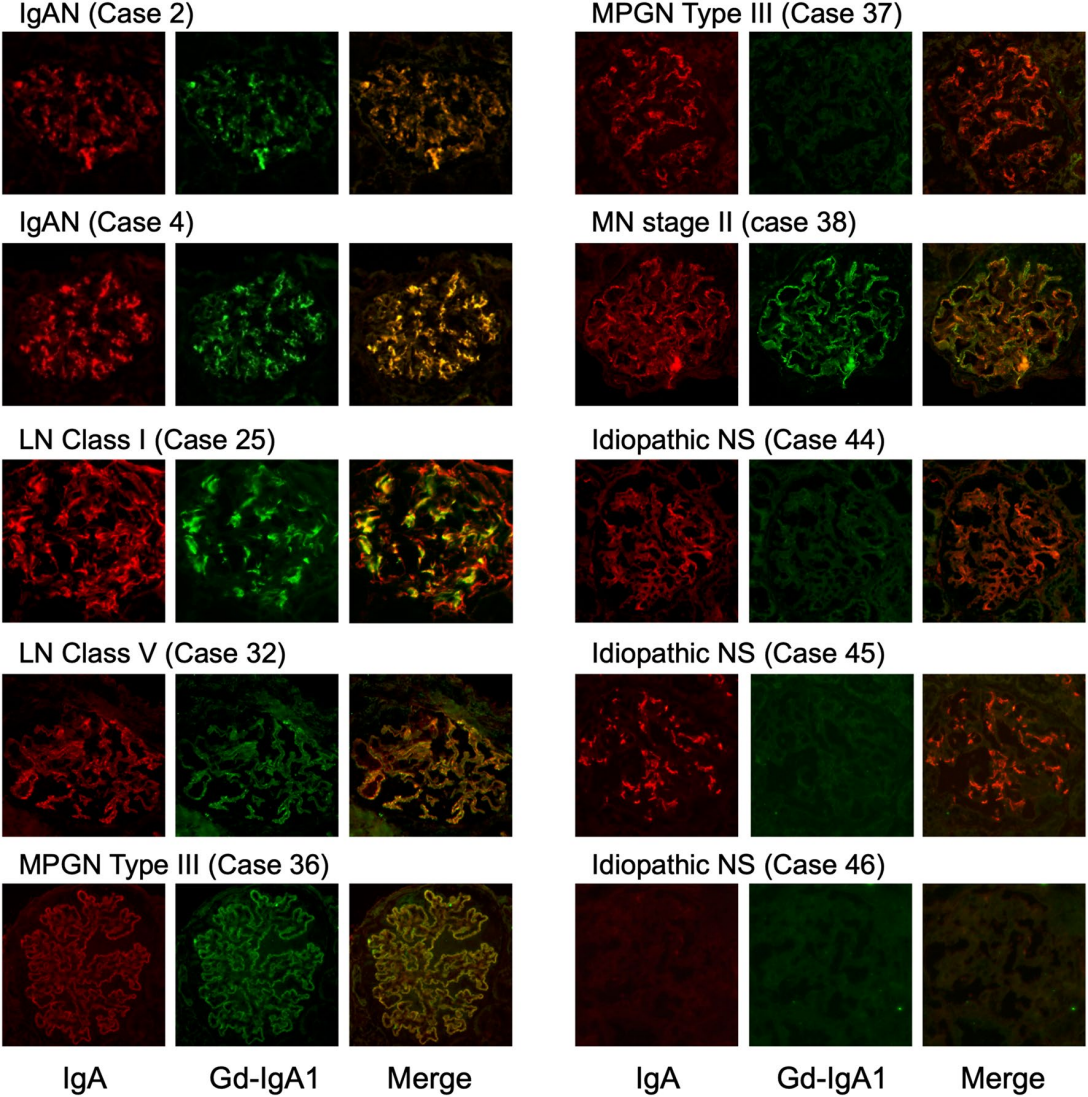
Double-immunofluorescence staining for IgA and Gd-IgA1. Double-immunofluorescence staining of frozen sections of biopsies from pediatric patients with glomerular diseases. First column, IgA staining; second column, Gd-IgA1 monoclonal antibody (KM55) staining; third column, merged images. All images ×200. Case numbers refer to cases listed in Supplementary Table 1. In patients with IgAN (cases 2 and 4) and LN class I (case 25), glomerular Gd-IgA1 deposition was detected, localized in the mesangial region with IgA. In patients with LN class V (case 32), MPGN type III (case 36), and MN stage II (case 38), both Gd-IgA1 and IgA findings were positive along the capillary wall. A patient with MPGN type III (case 37) showed only IgA-positive findings. In two patients with idiopathic NS (cases 44 and 45), Gd-IgA1 findings were negative, but IgA was clearly localized in the global mesangial area. Both Gd-IgA and IgA findings were negative in a patient with idiopathic NS (case 46). *IgAN* IgA nephropathy, *LN* lupus nephritis, *MPGN* membranoproliferative glomerulonephritis, *MN* membranous nephropathy, *NS* nephrotic syndrome.

The numbers of patients with IgA- and Gd-IgA1-positive findings are shown in Table 1. Sample immunofluorescence staining of IgA and Gd-IgA1 is shown in Fig. 1. The results for all patients are shown in Supplementary Fig. 1; the characteristics, conventional immunofluorescence, and clinical information are shown in Supplementary Table 1. Although most kidney specimens included only one glomerulus in the examined section, similar Gd-IgA1 staining results were obtained in all glomeruli when two or more were examined in a single specimen (Supplementary Table 1). Glomerular Gd-IgA1 was detected in all patients with IgAN and IgAV-N, regardless of disease grade. Among 17 patients with IgAN, light microscopy revealed diffuse mesangial proliferation in eight patients and focal mesangial proliferation in nine patients. Pathological findings of IgAV-N were categorized as International Study of Kidney Disease in Children (ISKDC) grade II in one patient and grade IIIb in five patients. Immunostaining results for two patients with IgAN are shown in Fig. 1. The immunostaining intensities differed between patients with IgAN and those with IgAV-N, but the pattern of Gd-IgA1 deposits was similar to that of IgA in the mesangial area in all patients. In addition, deposition of both IgA and Gd-IgA1 was detected in all patients with lupus nephritis. The lupus nephritis classification (2004 ISN/RPS) used in this study was as follows: class I (n = 3), II (n = 1), IIIA (n = 2), IV (n = 1), and V (n = 2). Gd-IgA1 deposits were localized in a pattern similar to that of IgA in mesangial areas in patients with lupus nephritis class I–IV and in capillary areas in patients with lupus nephritis class V. Gd-IgA1 deposition was also observed in three of four patients with membranoproliferative glomerulonephritis (MPGN) and one patient with primary membranous nephropathy accompanied by IgA deposition. Gd-IgA1 deposition was not observed in patients with other glomerular diseases involving mesangial IgA deposition, including idiopathic nephrotic syndrome (n = 6), oligomeganephronia (n = 2), Alport syndrome (n = 1), dense deposit disease (n = 1), and crescentic glomerulonephritis (n = 1). Both glomerular IgA and Gd-IgA1 findings were negative in patients with idiopathic nephrotic syndrome (n = 5), MPGN (n = 1), membranous nephropathy (n = 1), oligomeganephronia (n = 1), Alport syndrome (n = 1), C3 glomerulonephritis (n = 1), poststreptococcal acute glomerulonephritis (n = 1), and hemolytic uremic syndrome (n = 1).

**Table 1:** Numbers of patients with IgA-positive and Gd-IgA1-positive disease.

Disease	Number of cases (n)	Glomerular deposition	
		IgA (+)	Gd-IgA1 (+)
IgA nephropathy	17	17	17
IgA vasculitis with nephritis	6	6	6
Lupus nephritis	9	9	9
MPGN	5	4	3
Membranous nephropathy	2	1	1
Idiopathic nephrotic syndrome	11	6	0
Oligomeganephronia	3	2	0
Alport syndrome	2	1	0
Dense deposit disease	1	1	0
Crescentic glomerulonephritis	1	1	0
C3 glomerulonephritis	1	0	0
PSAGN	1	0	0
Hemolytic uremic syndrome	1	0	0

*MPGN* membranoproliferative glomerulonephritis, *PSAGN* poststreptococcal acute glomerulonephritis.

## Discussion

This study provides the first results of glomerular Gd-IgA1 staining using KM55 in pediatric patients. Gd-IgA1 staining results were positive in patients with IgAN and IgAV-N, as well as in patients with lupus nephritis, membranous nephropathy, and MPGN accompanied by IgA deposition. In contrast, no Gd-IgA1 deposits were observed in any patients with non-immune related glomerulonephritis accompanied by incidental glomerular IgA deposits.

The current results regarding pediatric patients with IgAN and IgAV-N are consistent with previous reports involving adult patients^7–10^. The anti-Gd-IgA1 monoclonal antibody KM55 was produced to detect serum Gd-IgA1 and glomerular Gd-IgA deposition in renal biopsy specimens using a novel ELISA approach. It has been proposed that IgAN and IgAV-N exhibit a shared pathogenesis involving Gd-IgA1 and Gd-IgA1-specific IgG autoantibodies^11,12^. Our results support this hypothesis. Furthermore, no Gd-IgA1 deposits were observed in patients with idiopathic nephrotic syndrome, oligomeganephronia, Alport syndrome, dense deposit disease, or crescentic glomerulonephritis accompanied by IgA deposition; however, only one or two specimens of some diseases were included in this study. IgA is not generally related to the mechanism of onset in these diseases, but incidental IgA deposition may occur even in healthy individuals^13,14^. Some patients in the present study showed IgA-positive findings, but Gd-IgA1 findings were negative in those patients, suggesting that KM55 has the potential to distinguish incidental IgA deposition. In case 45 in the current study (shown in Fig. 1; a patient with frequent relapsing nephrotic syndrome), renal biopsy was performed to examine cyclosporine nephrotoxicity. At the time of renal biopsy, urinalysis showed no proteinuria or hematuria during treatment with cyclosporine, whereas pathological findings revealed mild mesangial proliferation with IgA-dominant deposition in the mesangial area, similar to the findings for IgAN. It may thus be difficult to distinguish between IgAN and incidental IgA deposits by conventional pathological findings alone in such instances; KM55 may contribute to an accurate diagnosis and correct treatment.

The novel finding in the current study was that glomerular Gd-IgA1 deposition was observed at a similar intensity to that of IgA deposition in pediatric patients with lupus nephritis, primary membranous nephropathy, and MPGN. This result was inconsistent with previous reports involving adult patients. Suzuki et al. reported that Gd-IgA1 findings were negative in all adult patients with lupus nephritis (n = 7), primary membranous nephropathy (n = 1), and MPGN (n = 1) with IgA deposition^7^; furthermore, Cassol et al. demonstrated that Gd-IgA1 staining intensity was significantly lower in patients with lupus nephritis compared with patients with IgAN, while Gd-IgA1 findings were completely negative in five of eight patients with lupus nephritis^8^. However, the current study clearly showed Gd-IgA1-positive findings in these patients. There are several possible reasons for this discrepancy. First, the disease pathogenicity may differ between adults and children; thus, this study could provide new insight into the involvement of Gd-IgA1 in the pathogenicity of these diseases, which may explain glomerular Gd-IgA1 deposition in children. The multi-hit theory for onset of IgAN suggests that genetic or environmental factors are related to the overproduction of Gd-IgA1 and Gd-IgA1-specific IgG; these factor may lead to the formation of an immunocomplex and mesangial Gd-IgA1 deposition^4^. Recent genome-wide association studies identified several common susceptibility genes for systemic lupus erythematosus and IgAN^15,16^, suggesting that they may share common disease mechanisms. In contrast, genetic variation in *PLA2R1* has been associated with membranous nephropathy; a risk allele at *HLA-DQA1* has also been associated with increased risks of various autoimmunity-associated diseases, but not IgAN^17^. Furthermore, ELISA analysis of the antigenic specificity of extracted IgG showed that IgG extracted from immune deposits in patients with IgAN reacted with Gd-IgA1, while IgG extracted from patients with lupus nephritis and membranous nephropathy immune deposits did not^18^. Further studies are therefore needed to clarify the involvement of Gd-IgA1 in the pathogenesis of lupus nephritis, primary membranous nephropathy, and MPGN, especially in children. Second, a lack of specificity of KM55 should be considered. Patients with IgAN, IgAV-N, lupus nephritis, membranous nephropathy, and immunocomplex-mediated MPGN all demonstrate glomerular immunocomplex deposits as a key factor in the pathogenesis and progression of the respective diseases. Considering the pathogenicity of these diseases, KM55 might recognize IgA-related immunocomplexes in a non-specific manner. However, in the current study, one of four patients with MPGN accompanied by IgA deposition exhibited Gd-IgA1-negative findings (case 37, Fig. 1). The disease in this patient was also considered to be immunocomplex-mediated, because codominant IgG and C3 deposition was observed along the capillary wall; however, IgA deposition was detected segmentally and slightly in the mesangial region, not along the capillary wall as observed for IgG. Therefore, the IgA deposition in this patient might have occurred irrespective of the disease mechanism, and KM55 could thus distinguish incidental IgA deposition.

Differences between the current and previous reports might also have resulted from different staining procedures. We used frozen sections of biopsy specimens, while other studies used paraffin-embedded sections. Immunostaining of frozen sections could be associated with stronger antigenicity in some instances, leading to potential discrepancies between positive or negative staining results in patients with lupus nephritis, membranous nephropathy, or MPGN. Although this was a possible limitation of our analysis, the use of frozen sections could also have been beneficial, because our procedure did not require antigen retrieval and was able to be performed more easily, compared with analysis of paraffin-embedded sections.

This study had some limitations. First, the number of patients was relatively small. For patients with some diseases, the specificity of Gd-IgA1 staining was evaluated by using only one or two biopsy specimens. Thus, there might have been bias in the results regarding these diseases. Additional data are needed to clarify the diagnostic value of KM55. In addition, few glomeruli were observed in each specimen, as shown in Supplementary Table 1. Second, we could not analyze serum Gd-IgA1 levels or the relationship between serum levels of Gd-IgA1 and glomerular Gd-IgA1 deposition intensity, because an insufficient number of blood samples was obtained. Third, as noted above, the staining procedure differed from the method used in previous studies of Gd-IgA1 staining.

In conclusion, glomerular Gd-IgA1 deposition was detected in patients with IgAN and IgAV-N, as well as in pediatric patients with lupus nephritis, primary membranous nephropathy, and immunocomplex-mediated MPGN. These findings could offer new insights into the involvement of Gd-IgA1 in the pathogenicity of these diseases. In addition, KM55 might recognize part of the immunocomplex involved in this pathogenicity, and may have the ability to detect incidental IgA deposition in patients with other glomerular diseases.

## Methods

### Study design and participants

This retrospective, multicenter study enrolled 60 pediatric patients with glomerular diseases who underwent renal biopsies at Kobe University Hospital (n = 44), Osaka City General Hospital (n = 7), Wakayama Medical University (n = 4), Takatsuki General Hospital (n = 3), and Kobe Children’s Hospital (n = 2) between July 2007 and January 2020. The renal biopsies were performed by pediatric nephrologists at each institute for various clinical indications. The patients’ electronic medical records were reviewed to obtain their clinical information. The patients’ characteristics are shown in Table 2 and Supplementary Table 1. IgA staining results were also positive for some patients with non-immune related glomerulonephritis (Table 1, Supplementary Table 1). There were 28 male patients and 32 female patients; the mean age of the patients at renal biopsy was 10.5 years (range, 2–18 years).

**Table 2:** Characteristics of enrolled patients.

Disease	Number of cases (n)	Female (n)	Age (years)	Proteinuria (g/g Cr)	Serum alb (g/dL)	eGFR (ml/min/1.73 m^2^)
IgA nephropathy	17	9	12 (7–15)	1.1 (0.0–4.9)	3.8 (3.1–4.9)	107 (79–130)
IgA vasculitis with nephritis	6	2	5 (5–15)	3.8 (0.2–15.9)	3.0 (1.7–4.4)	115 (103–139)
Lupus nephritis	9	7	13 (8–18)	0.7 (0.1–5.5)	3.3 (2.7–4.5)	108 (63–198)
MPGN	5	2	13 (5–16)	1.5 (0.2–2.8)	3.6 (1.1–4.6)	122 (37–201)
Membranous nephropathy	2	0	5 (3–7)	1.9 (1.8–2.1)	3.4 (3.2–3.5)	119 (110–128)
Idiopathic nephrotic syndrome	11	6	7 (2–16)	0.1 (0.0–29.1)	4.1 (1.7–4.6)	109 (87–142)
Oligomeganephronia	3	1	15 (12–16)	0.7 (0.3–1.2)	4.2 (4.1–4.5)	62 (58–66)
Alport syndrome	2	2	3.5 (3–4)	0.3 (0.2–0.4)	4.4 (4.2–4.5)	134 (126–142)
Dense deposit disease	1	0	10	1.4	3.6	78
Crescentic glomerulonephritis	1	1	17	3.3	4.0	102
C3 glomerulonephritis	1	0	12	0.1	4.6	94
PSAGN	1	1	8	10.8	1.9	42
Hemolytic uremic syndrome	1	1	4	1.1	4.9	55

Data were obtained at the time of renal biopsy and are presented as median (range).

*alb* albumin, *eGFR* estimated glomerular filtration rate, *MPGN* membranoproliferative glomerulonephritis, *PSAGN* poststreptococcal acute glomerulonephritis.

### Clinical and pathological definitions

The estimated glomerular filtration rate (GFR) was calculated using the creatinine-based equation for Japanese children and adolescents^19^. Histological sections were analyzed by pathologists at each institute. Immunofluorescence intensity was analyzed in frozen sections and defined as generally negative/trace (0.5), 1+, 1/2+, 2+, 2/3+, and 3+.

### Immunohistochemical staining of Gd-IgA1

Double-immunofluorescence staining of IgA and Gd-IgA1 was performed using frozen sections of renal biopsy specimen. Frozen sections (3 μm) were fixed in acetone for 10 min and washed several times with 10% phosphate-buffered saline (PBS). The samples were then blocked with 10% goat serum at room temperature for 60 min, washed with 10% PBS, and incubated with rat monoclonal anti-human Gd-IgA1 antibody (100 μg/mL; KM55, Immuno-Biological Laboratories, Fujioka, Japan) at 37 °C for 60 min. After samples had been washed several times in 10% PBS, they were incubated with polyclonal rabbit anti-human IgA antibody (100 μg/mL; Dako Envision FLEX-IgA, Dako Japan, Tokyo, Japan) at 37 °C for 60 min, washed several times with 10% PBS, and sealed at 37 °C for 30 min with Alexa Fluor 488-conjugated goat anti-rat IgG antibody (1:100; Life Technologies, CA, USA) and Alexa Fluor 546-conjugated goat anti-rabbit IgG antibody (1:100; Life Technologies). The stained slides were then viewed using a fluorescence microscope (BZ-X710; KEYENCE, Osaka, Japan) and the intensity of glomerular Gd-IgA1 was scored from 0 to 3+, with 0 and trace (0.5+) regarded as negative staining and ≥ 1+ regarded as positive staining.

### Ethical approval

All procedures performed in studies involving human participants were carried out in accordance with the ethical standards of the Institutional Review Board of Kobe University Graduate School of Medicine (IRB approval number: B190137), Osaka City General Hospital, Wakayama Medical University, Takatsuki General Hospital and Kobe Children’s Hospital, as well as with the 1964 Helsinki Declaration and its later amendments or comparable ethical standards. Comprehensive written informed consent was obtained from all individual participants and/or guardians included in the study to use residual biopsied kidney samples and patients’ clinical data.

## Supplementary information

Supplementary information.

## Data Availability

Deidentified data and materials are available from the corresponding author on reasonable request.

## References

[1] Kamei K (2016). Proteinuria during follow-up period and long-term renal survival of childhood IgA nephropathy. PLoS ONE.

[2] Allen AC (2001). Mesangial IgA1 in IgA nephropathy exhibits aberrant O-glycosylation: Observations in three patients. Kidney Int..

[3] Moldoveanu Z (2007). Patients with IgA nephropathy have increased serum galactose-deficient IgA1 levels. Kidney Int..

[4] Suzuki H (2011). The pathophysiology of IgA nephropathy. J. Am. Soc. Nephrol..

[5] Lau KK (2007). Serum levels of galactose-deficient IgA in children with IgA nephropathy and Henoch-Schonlein purpura. Pediatr. Nephrol..

[6] Yasutake J (2015). Novel lectin-independent approach to detect galactose-deficient IgA1 in IgA nephropathy. Nephrol. Dial. Transplant..

[7] Suzuki H (2018). IgA nephropathy and IgA vasculitis with nephritis have a shared feature involving galactose-deficient IgA1-oriented pathogenesis. Kidney Int..

[8] Cassol CA (2019). Immunostaining for galactose-deficient immunoglobulin A is not specific for primary immunoglobulin A nephropathy. Nephrol. Dial. Transplant.

[9] Wada Y (2018). Clinical significance of serum and mesangial galactose-deficient IgA1 in patients with IgA nephropathy. PLoS ONE.

[10] Zhang K (2019). Clinical significance of galactose-deficient IgA1 by KM55 in patients with IgA nephropathy. Kidney Blood Press. Res..

[11] Kiryluk K (2011). Aberrant glycosylation of IgA1 is inherited in both pediatric IgA nephropathy and Henoch-Schonlein purpura nephritis. Kidney Int..

[12] Suzuki H, Moldoveanu Z, Julian BA, Wyatt RJ, Novak J (2019). Autoantibodies specific for galactose-deficient IgA1 in IgA vasculitis with nephritis. Kidney Int. Rep..

[13] Suzuki K (2003). Incidence of latent mesangial IgA deposition in renal allograft donors in Japan. Kidney Int..

[14] Sinniah R (1983). Occurrence of mesangial IgA and IgM deposits in a control necropsy population. J. Clin. Pathol..

[15] Zhou XJ (2014). Brief Report: Identification of MTMR3 as a novel susceptibility gene for lupus nephritis in northern Han Chinese by shared-gene analysis with IgA nephropathy. Arthritis Rheumatol..

[16] Kiryluk K (2014). Discovery of new risk loci for IgA nephropathy implicates genes involved in immunity against intestinal pathogens. Nat. Genet..

[17] Sekula P (2017). Genetic risk variants for membranous nephropathy: Extension of and association with other chronic kidney disease aetiologies. Nephrol. Dial. Transplant.

[18] Rizk DV (2019). Glomerular immunodeposits of patients with IgA nephropathy are enriched for IgG autoantibodies specific for galactose-deficient IgA1. J. Am. Soc. Nephrol..

[19] Uemura O (2014). Creatinine-based equation to estimate the glomerular filtration rate in Japanese children and adolescents with chronic kidney disease. Clin. Exp. Nephrol..

