# Supplementary material for: Glomerular galactose-deficient IgA1 expression analysis in pediatric patients with glomerular diseases
Source: Sci Rep. 2020 Aug 20;10:14026. doi: 10.1038/s41598-020-71101-y (PMC7441388; doi:10.1038/s41598-020-71101-y)
Supplement: Supplementary file 1 — Supplementary information. [file 41598_2020_71101_MOESM1_ESM.pdf]

**Glomerular galactose-deficient IgA1 expression analysis in pediatric patients with glomerular diseases**

Shinya Ishiko, Tomoko Horinouchi, Rika Fujimaru, Yuko Shima, Hiroshi Kaito, Ryojiro Tanaka, Shingo Ishimori, Atsushi Kondo, Sadayuki Nagai, Yuya Aoto, Nana Sakakibara, China Nagano, Tomohiko Yamamura, Momoka Yoshimura, Koichi Nakanishi, Junya Fujimura, Naohiro Kamiyoshi, Hiroaki Nagase, Norishige Yoshikawa, Kazumoto Iijima, Kandai Nozu

Supplementary Figure

Results of double-immunofluorescence staining for IgA and Gd-Ig1 using frozen sections of biopsies from 60 pediatric patients with various glomerular diseases. First column, IgA staining; second column, Gd-IgA1 monoclonal antibody (KM55) staining; third column, merged images. IgAN; IgA nephropathy, IgAV-N; IgA vasculitis with nephritis, LN; lupus nephritis, MPGN; membranoproliferative glomerulonephritis, MN; membranous nephropathy, INS; idiopathic nephrotic syndrome, OMN; oligomeganephronia, DDD; dense deposit disease, GN; glomerulonephritis, PSAGN; poststreptococcal acute glomerulonephritis, HUS; hemolytic uremic syndrome

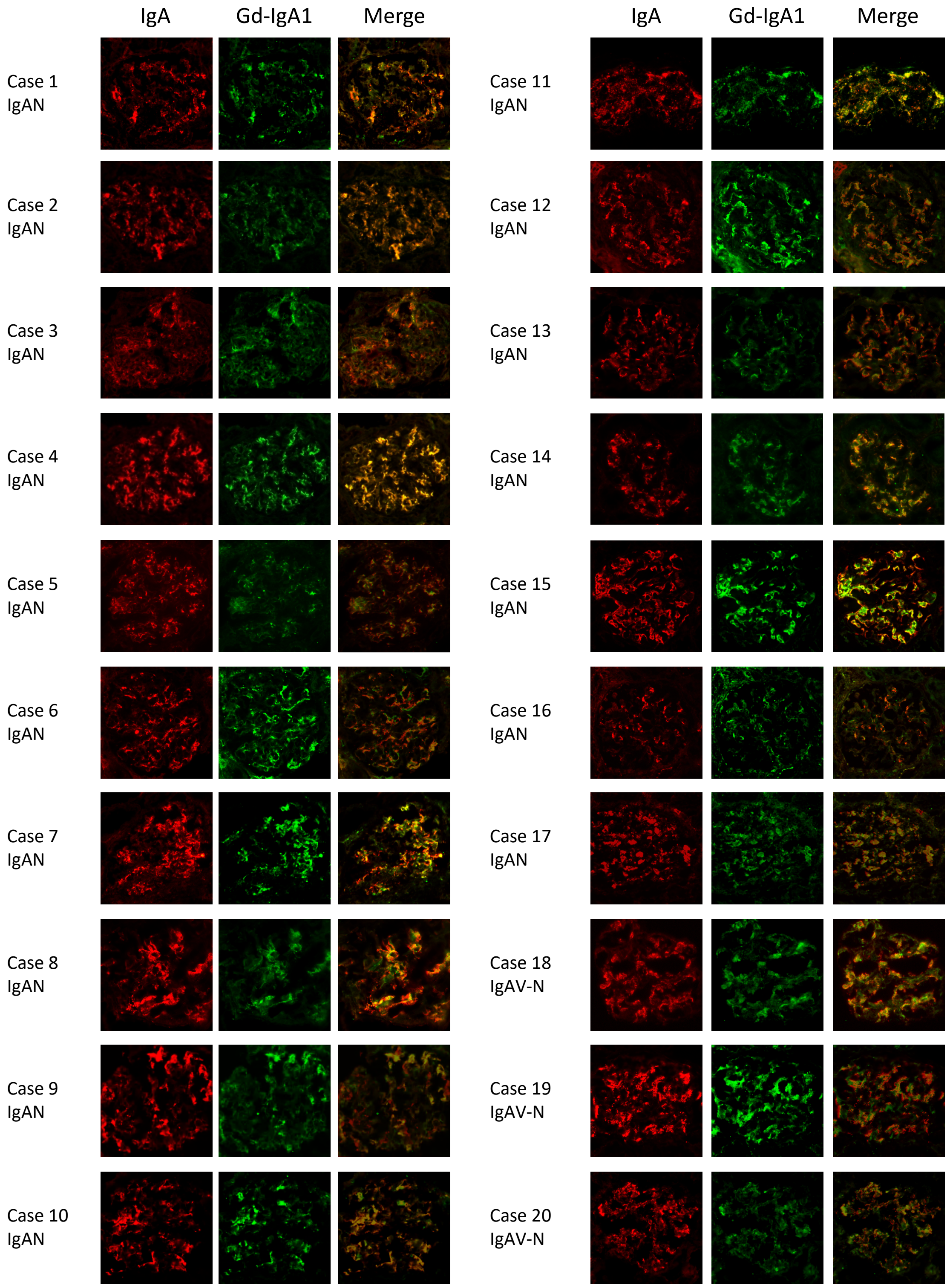

IgA
Gd-IgA1
Merge
Case 1 IgAN
Case 2 IgAN
Case 3 IgAN
Case 4 IgAN
Case 5 IgAN
Case 6 IgAN
Case 7 IgAN
Case 8 IgAN
Case 9 IgAN
Case 10 IgAN
Case 11 IgAN
Case 12 IgAN
Case 13 IgAN
Case 14 IgAN
Case 15 IgAN
Case 16 IgAN
Case 17 IgAN
Case 18 IgAV-N
Case 19 IgAV-N
Case 20 IgAV-N

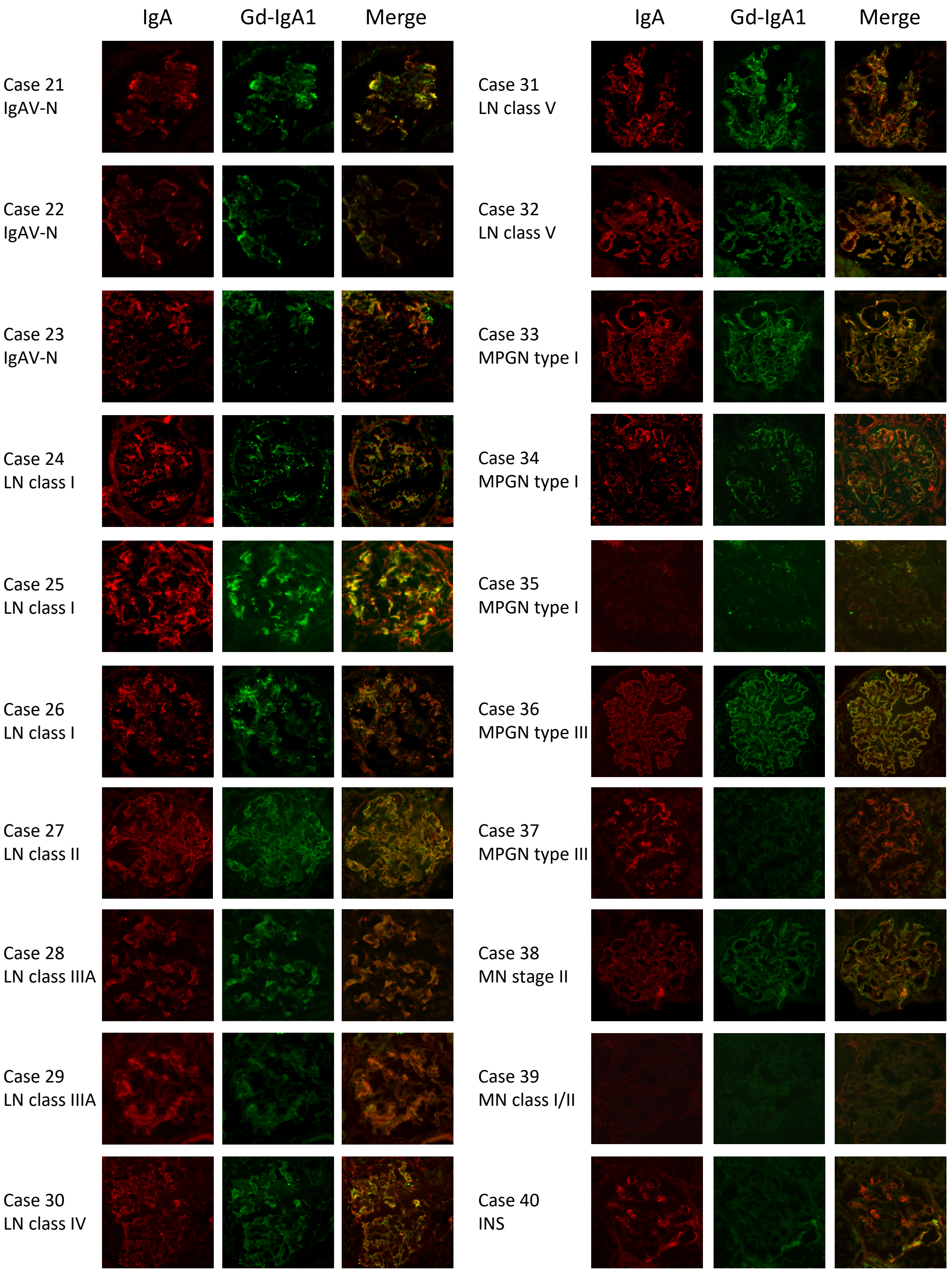
IgA
Gd-IgA1
Merge
IgA
Gd-IgA1
Merge
Case 21
IgAV-N
Case 22
IgAV-N
Case 23
IgAV-N
Case 24
LN class I
Case 25
LN class I
Case 26
LN class I
Case 27
LN class II
Case 28
LN class IIIA
Case 29
LN class IIIA
Case 30
LN class IV
Case 31
LN class V
Case 32
LN class V
Case 33
MPGN type I
Case 34
MPGN type I
Case 35
MPGN type I
Case 36
MPGN type III
Case 37
MPGN type III
Case 38
MN stage II
Case 39
MN class I/II
Case 40
INS

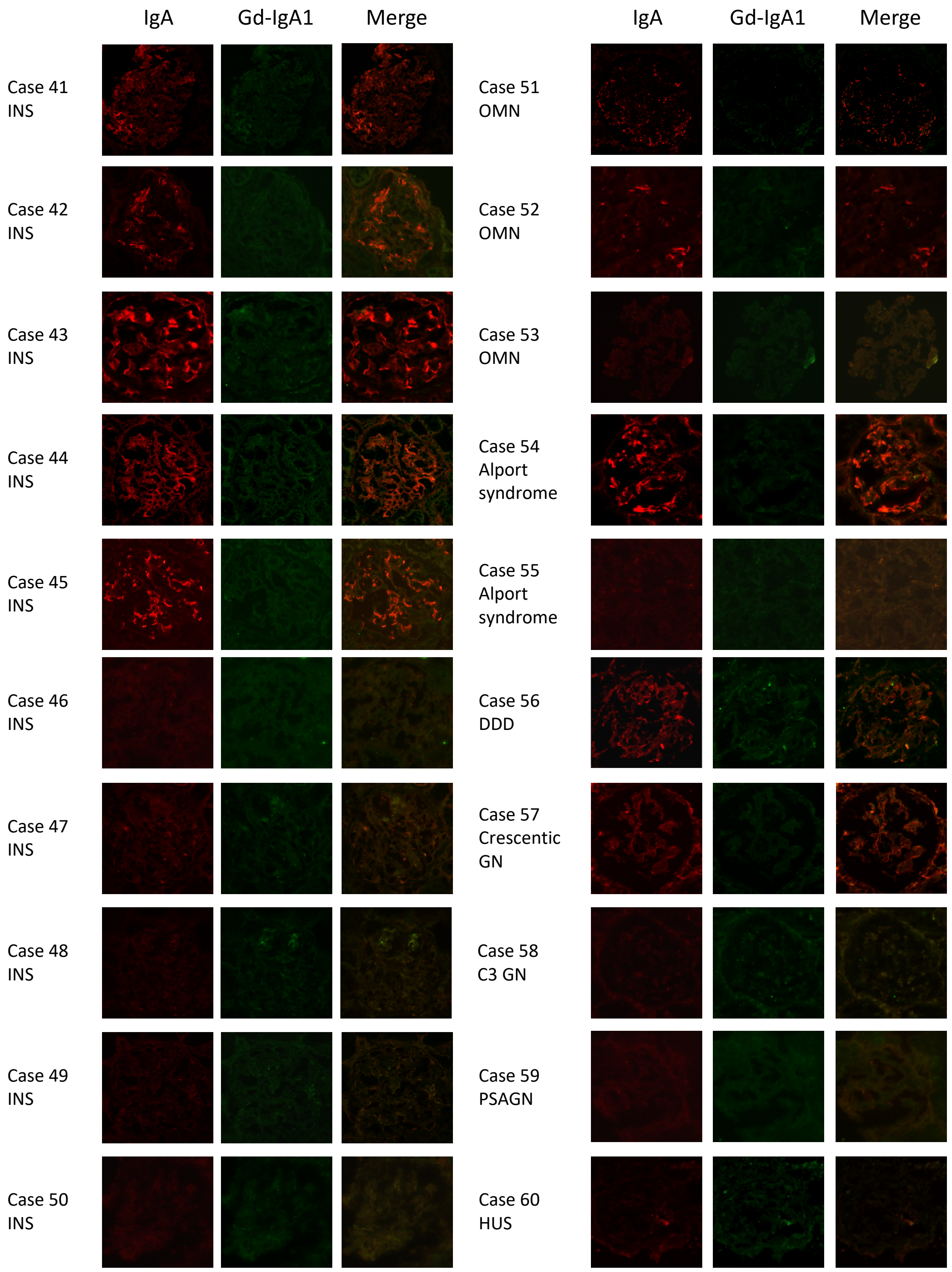
IgA
Gd-IgA1
Merge
IgA
Gd-IgA1
Merge
Case 41 INS
Case 42 INS
Case 43 INS
Case 44 INS
Case 45 INS
Case 46 INS
Case 47 INS
Case 48 INS
Case 49 INS
Case 50 INS
Case 51 OMN
Case 52 OMN
Case 53 OMN
Case 54 Alport syndrome
Case 55 Alport syndrome
Case 56 DDD
Case 57 Crescentic GN
Case 58 C3 GN
Case 59 PSAGN
Case 60 HUS

Supplementary Table

| Case no. | Age (years) | Sex | Diagnosis | Number of glomeruli | Glomerular deposition | | | | | | Proteinuria (g/g • Cr) | RBC in urine (/HPF) | eGFR (ml/min/ 1.73 m^2) | Serum albumin (g/dL) |
|---|---|---|---|---|---|---|---|---|---|---|---|---|---|---|
| | | | | | Gd-IgA1 | IgA | IgG | IgM | C1q | C3 | | | | |
| 1 | 9 | F | IgAN (Diffuse) | 3 | 2 | 2 | 1 | 0 | 0 | 0 | 0.50 | 20-29 | 105 | 4.4 |
| 2 | 9 | M | IgAN (Diffuse) | 2 | 2.5 | 2.5 | 2 | 1 | 0 | 1 | 4.92 | 10-19 | 107 | 3.3 |
| 3 | 10 | F | IgAN (Diffuse) | 1 | 1 | 1.5 | 0.5 | 1.5 | 1 | 1 | 1.01 | 30-49 | 130 | 3.3 |
| 4 | 12 | M | IgAN (Diffuse) | 1 | 2 | 2 | 1 | 1 | 0 | 1 | 1.13 | >100 | 126 | 3.7 |
| 5 | 12 | F | IgAN (Diffuse) | 2 | 1 | 3 | 1 | 1 | 0 | 2 | 1.46 | >100 | 108 | 3.3 |
| 6 | 14 | F | IgAN (Diffuse) | 1 | 1 | 2 | 1 | 1 | 0 | 1 | 1.05 | >100 | 110 | 3.7 |
| 7 | 14 | F | IgAN (Diffuse) | 2 | 2 | 2 | 1 | 2 | 1 | 3 | 1.22 | 20-29 | 116 | 3.6 |
| 8 | 15 | M | IgAN (Diffuse) | 1 | 1 | 1.5 | 1 | 1 | 0 | 1 | 0.03 | 1-4 | 79 | 4.9 |
| 9 | 7 | M | IgAN (Focal) | 1 | 2 | 2 | 0.5 | 1 | 0 | 0 | 1.81 | 50-99 | 81 | 3.9 |
| 10 | 7 | F | IgAN (Focal) | 1 | 1 | 1.5 | 1 | 0.5 | 0.5 | 1 | 1.60 | 30-49 | 127 | 3.1 |
| 11 | 10 | F | IgAN (Focal) | 2 | 2 | 2 | 1 | 1 | 0 | 1 | 0.98 | >100 | 127 | 4.3 |
| 12 | 11 | M | IgAN (Focal) | 2 | c2/m2 | c2/m2 | 1 | 1 | 1 | 0.5 | 3.80 | >100 | 104 | 3.8 |
| 13 | 11 | M | IgAN (Focal) | 1 | 2 | 3 | 1 | 0.5 | 0 | 1 | 1.20 | >100 | 102 | 4.0 |
| 14 | 12 | M | IgAN (Focal) | 1 | 2 | 2.5 | 1 | 0 | 0 | 2 | 0.40 | >100 | 101 | 3.8 |
| 15 | 13 | F | IgAN (Focal) | 1 | 2.5 | 2.5 | 2 | 1 | 0 | 1 | 0.91 | 50-99 | 98 | 3.9 |
| 16 | 14 | M | IgAN (Focal) | 1 | 1 | 2 | 1 | 1 | 1 | 1 | 0.44 | 20-29 | 130 | 4.1 |
| 17 | 14 | F | IgAN (Focal) | 2 | 2 | 3 | 0 | 1 | 0 | 1 | 0.17 | >100 | 107 | 4.2 |
| 18 | 5 | F | IgAV-N (ISKDC II) | 2 | 2 | 3 | 1 | 1 | 0 | 1 | 15.9 | 10-19 | 125 | 3.1 |
| 19 | 5 | M | IgAV-N (ISKDC IIIb) | 1 | 2 | 3 | 2 | 1 | 0 | 0 | 3.23 | 5-9 | 113 | 2.8 |
| 20 | 5 | M | IgAV-N (ISKDC IIIb) | 3 | 2 | 3 | 2 | 0 | 0 | 0 | 5.81 | >100 | 103 | 2.4 |
| 21 | 5 | F | IgAV-N (ISKDC IIIb) | 5 | c1/m2 | c1/m2 | 1 | 1 | 0 | 1 | 2.70 | >100 | 139 | 3.3 |
| 22 | 9 | M | IgAV-N (ISKDC IIIb) | 1 | 1 | 2.5 | 1 | 0 | 0 | 1 | 4.27 | >100 | 108 | 1.7 |
| 23 | 15 | M | IgAV-N (ISKDC IIIb) | 1 | 1 | 2 | 1 | 0 | 0 | 2 | 0.20 | 10-19 | 116 | 4.4 |
| 24 | 8 | F | LN (class I) | 2 | 1 | 1 | 1s/1d | 1 | 1 | 1 | 0.67 | 1-4 | 198 | 3.6 |
| 25 | 13 | F | LN (class I) | 2 | 1.5 | 1.5 | 1.5 | 2 | 2 | 2 | 0.19 | 1-4 | 106 | 3.5 |
| 26 | 15 | F | LN (class I) | 1 | 2 | 2 | 1 | 2 | 3 | 3 | 0.10 | <1 | 112 | 3.6 |
| 27 | 13 | M | LN (class II) | 7 | 1 | 1 | c2/m2 | 1 | 2 | c3/m3 | 0.20 | 20-29 | 102 | 3.2 |

| | | | | | | | | | | | | | | |
|---|---|---|---|---|---|---|---|---|---|---|---|---|---|---|
| 28 | 14 | F | LN (class IIIA) | 2 | 3 | 3 | 3 | 2 | 3 | 3 | 3.69 | 1-4 | 113 | 3.2 |
| 29 | 14 | F | LN (class IIIA) | 1 | 1 | 1 | 0.5 | 2 | 2 | 3 | 1.10 | 5-9 | 100 | 3.3 |
| 30 | 11 | M | LN (class IV) | 1 | 1 | 1 | c2 | 0 | c2/m2 | 1 | 3.60 | 30-49 | 63 | 2.7 |
| 31 | 12 | F | LN (class V) | 2 | c1 | c1 | c2 | c1 | c0.5 | c2 | 5.45 | 1-4 | 146 | 2.9 |
| 32 | 18 | F | LN (class V) | 1 | c1 | c1 | c2 | 0 | c1 | c1 | 0.10 | 1-4 | 108 | 4.5 |
| 33 | 5 | M | MPGN type I | 4 | c1 | c1 | 1s | 1 | 1 | c1/m2 | 0.48 | 10-19 | 150 | 3.6 |
| 34 | 16 | F | MPGN type I | 2 | c1 | c1 | c2/m2 | c2/m2 | c2/m2 | c3/m3 | 0.15 | 1-4 | 122 | 4.6 |
| 35 | 13 | M | MPGN type I | 5 | 0 | 0 | c1/m1 | c1/m1 | 1 | c1/m3 | 2.00 | >100 | 37 | 3.2 |
| 36 | 14 | M | MPGN type III | 1 | c2 | c2 | c3 | 1 | c3 | c2 | 2.79 | >100 | 201 | 1.1 |
| 37 | 11 | F | MPGN type III | 4 | 0 | 1 | c2/m2 | 0 | 1 | 1 | 1.53 | 5-9 | 106 | 3.9 |
| 38 | 3 | M | Primary MN (stage II) | 5 | c1 | c1 | c1/2 | 0 | c1 | 0 | 1.79 | 30-49 | 111 | 3.5 |
| 39 | 7 | M | Primary MN (stage I/II) | 1 | 0 | 0 | c1 | 0 | c1 | 0 | 2.05 | 1-4 | 128 | 3.2 |
| 40 | 5 | M | INS (FRNS) | 3 | 0 | 1 | 1 | 1 | n/d | 1 | 0.05 | <1 | 134 | 3.7 |
| 41 | 7 | F | INS (SRNS) | 1 | 0 | 1 | 0 | 0 | 1 | 0 | 0.08 | 5-9 | 109 | 3.2 |
| 42 | 7 | M | INS (FRNS) | 1 | 0 | 1 | 0 | 0 | n/d | 0 | 0.06 | <1 | 108 | 4.6 |
| 43 | 8 | F | INS | 2 | 0 | 1 | 1 | 1s | 1 | 0 | 29.1 | 5-10 | 106 | 1.7 |
| 44 | 8 | M | INS (FRNS) | 2 | 0 | 1 | 0 | 1 | 1 | 1 | 0.03 | <1 | 87 | 4.2 |
| 45 | 10 | M | INS (FRNS) | 4 | 0 | 2 | 1 | 1 | n/d | 1 | 0.02 | <1 | 105 | 3.8 |
| 46 | 2 | F | INS (SRNS) | 1 | 0 | 0 | 0 | 0 | 0 | 0 | 0.51 | <1 | 118 | 2.6 |
| 47 | 4 | F | INS (FRNS) | 1 | 0 | 0 | 0 | 0 | 0 | 0 | 0.06 | 1-4 | 142 | 4.1 |
| 48 | 5 | M | INS (FRNS/SDNS) | 2 | 0 | 0 | 0 | 0 | 0 | 0 | 0.03 | <1 | 117 | 4.4 |
| 49 | 7 | F | INS (FRNS) | 3 | 0 | 0 | 0 | 0.5 | 0 | 0 | 0.09 | <1 | 95 | 4.2 |
| 50 | 16 | F | INS (FRNS/SDNS) | 1 | 0 | 0 | 0 | 0 | 0 | 0 | 0.02 | 30-49 | 114 | 4.5 |
| 51 | 15 | M | Oligomeganephronia | 2 | 0 | 1 | 1 | 1 | 0 | 1 | 0.70 | <1 | 62 | 4.5 |
| 52 | 16 | F | Oligomeganephronia | 1 | 0 | 1 | 0.5 | 0.5 | 0 | 0 | 1.20 | <1 | 58 | 4.1 |
| 53 | 12 | M | Oligomeganephronia | 1 | 0 | 0 | 0 | 1 | 0 | 0 | 0.29 | <1 | 66 | 4.2 |
| 54 | 4 | F | Alport syndrome | 3 | 0 | 1 | 0 | 0 | 0 | 0 | 0.21 | >100 | 142 | 4.2 |
| 55 | 3 | F | Alport syndrome | 2 | 0 | 0 | 0 | 1 | 0 | 0 | 0.35 | >100 | 126 | 4.5 |
| 56 | 10 | M | Dense deposit disease | 2 | 0 | c1 | c1/m1 | c1 | 1 | c3/m3 | 1.35 | 30-49 | 78 | 3.6 |
| 57 | 17 | F | Crescentic GN | 2 | 0 | c1/m1 | c1/m1 | c2/m2 | m2 | 2 | 3.25 | 1-4 | 102 | 4.0 |
| 58 | 12 | M | C3 GN | 4 | 0 | 0 | 0 | 0 | 0 | 3 | 0.12 | 5-9 | 94 | 4.6 |

| | | | | | | | | | | | | | | |
|---|---|---|---|---|---|---|---|---|---|---|---|---|---|---|
| 59 | 8 | F | PSAGN | 1 | 0 | 0 | 0 | 0 | 1 | 2 | 10.8 | 50-99 | 42 | 1.9 |
| 60 | 4 | F | HUS | 1 | 0 | 0 | 0 | 0 | 0 | 0 | 1.11 | 1-4 | 55 | 4.9 |

RBC; Red Blood Cell, HPF; high-power field, eGFR; estimated glomerular filtration rate, IgAN; IgA nephropathy, IgAV-N; IgA vasculitis with nephritis, LN; lupus nephritis, MPGN; membranoproliferative glomerulonephritis, MN; membranous nephropathy, INS; idiopathic nephrotic syndrome, FRNS; frequent relapsing nephrotic syndrome, SDNS; steroid-resistant nephrotic syndrome, GN; glomerulonephritis, PSAGN; poststreptococcal acute glomerulonephritis, HUS; hemolytic uremic syndrome. IgAN was classified into two types: diffuse IgAN showed diffuse mesangial proliferation and focal IgAN showed focal mesangial proliferation by light microscopic findings. Immunofluorescence intensity was analyzed in frozen sections and defined as generally negative (-) to trace (0.5), 1+, 1/2+, 2+, 2/3+ and 3+. 'c' indicates deposition along capillary walls; 'm' indicates deposition in the mesangial region; number alone indicates deposition in the mesangial region. Clinical data were taken at the time of renal biopsy. n/d indicates no date.
